# Production and efficacy of a low-cost recombinant pneumococcal protein polysaccharide conjugate vaccine

**DOI:** 10.1016/j.vaccine.2018.05.036

**Published:** 2018-06-18

**Authors:** Jenny A. Herbert, Emily J. Kay, Sian E. Faustini, Alex Richter, Sherif Abouelhadid, Jon Cuccui, Brendan Wren, Timothy J. Mitchell

**Affiliations:** a Institute of Microbiology and Infection, College of Medical and Dental Sciences, University of Birmingham, Birmingham, England, UK; b Department of Pathogen Molecular Biology, London School of Hygiene and Tropical Medicine, London, UK; c Institute of Immunology and Immunotherapy, College of Medical and Dental Sciences, University of Birmingham, Birmingham, England, UK; d Department of Immunology, Queen Elizabeth Hospital, Birmingham, UK; e University Hospitals Birmingham NHS Foundation Trust, Birmingham, UK

**Keywords:** *Streptococcus pneumoniae*, Conjugate vaccine, Protein glycan coupling technology, Glycoengineering, Pneumonia

## Abstract

*Streptococcus pneumoniae* is the leading cause of bacterial pneumonia. Although this is a vaccine preventable disease, *S. pneumoniae* still causes over 1 million deaths per year, mainly in children under the age of five. The biggest disease burden is in the developing world, which is mainly due to unavailability of vaccines due to their high costs. Protein polysaccharide conjugate vaccines are given routinely in the developed world to children to induce a protective antibody response against *S. pneumoniae*. One of these vaccines is Prevnar13, which targets 13 of the 95 known capsular types. Current vaccine production requires growth of large amounts of the 13 serotypes, and isolation of the capsular polysaccharide that is then chemically coupled to a protein, such as the diphtheria toxoid CRM_197,_ in a multistep expensive procedure. In this study, we design, purify and produce novel recombinant pneumococcal protein polysaccharide conjugate vaccines in *Escherichia coli*, which act as mini factories for the low-cost production of conjugate vaccines. Recombinant vaccine efficacy was tested in a murine model of pneumococcal pneumonia; ability to protect against invasive disease was compared to that of Prevnar13. This study provides the first proof of principle that protein polysaccharide conjugate vaccines produced in *E. coli* can be used to prevent pneumococcal infection. Vaccines produced in this manner may provide a low-cost alternative to the current vaccine production methodology.

## Highlights

• Pneumococcal glycoconjugate vaccines produced in *E. coli* are protective in mice.
• Protection correlates with opsonic antibody levels.
• This is a cheaper way to produce conjugate vaccines.

## 1 Introduction

*Streptococcus pneumoniae* (the pneumococcus) is the leading cause of bacterial pneumonia. The highest disease burden is observed in the developing world due to limited vaccine availability [1]. In the developed world, since the introduction of pneumococcal conjugate vaccines, *S. pneumoniae* disease burden in children has drastically reduced (PCV7/10/13) [2], [3]. Prevnar7 was the first pneumococcal conjugate vaccine produced and was introduced in the UK in 2006. Post introduction, invasive pneumococcal disease caused by vaccine serotypes dropped by 41% [2]. After introduction of Prevnar13 (PCV13) in 2010 invasive disease caused by the additional 6 serotypes dropped by 75% [4].

PCV13 is a component of the childhood vaccine schedule in the UK and is given to all children in a three-dose schedule at 2, 4 and 12 months of age. This vaccine targets the capsular polysaccharide surrounding the bacteria. PCV13 protects against the 13 (out of 95) pneumococcal serotypes found to be most prevalent in disease [5]. Polysaccharide alone is not immunogenic in children under 2 years of age, and does not produce a lasting immune response [6]. Conjugate vaccines work by coupling the polysaccharide component to a protein carrier [7], resulting in a protective T-cell dependent memory response [8]. This technology was first used for the production of a *Haemophilus influenzae* type B vaccine [9], followed by conjugate vaccines to prevent *Neisseria meningitidis* serogroup C [10], and subsequently pneumococcal infection [11]. Pneumococcal conjugate vaccines are the world’s best-selling vaccines, and in 2014 PCV13 sales produced revenues of £2.9 billion [12].

Although these glycoconjugate vaccines are very effective, there are some limitations to their use. Serotype distribution of disease causing isolates varies geographically [13]. The PCV7 vaccine serotypes are more prevalent in the western world, therefore this vaccine did not provide adequate protection against serotypes prevalent in developing countries. However, the introduction of an additional 6 serotypes in PCV13 includes the main disease-causing serotypes in the developing world [5], [13]. Serotype replacement remains a problem, and introduction of vaccines has resulted in increased incidence of disease from non-vaccine serotypes [2], [14], [15]. The most recent emerging serotypes (22F and 33F) are included in a new 15-valent vaccine preparation [16]. Finally, the high cost of conjugate vaccines means they are often not available to the poorest regions, which have the greatest disease burden.

The complex nature of the production process of the conjugate vaccine is one contributing factor to the high cost. Using standard methods, growth of large quantities of pathogenic pneumococci is required for isolation of the polysaccharide. Post purification the polysaccharide must then be chemically coupled to the carrier protein, in the case of PCV13 a diphtheria toxoid (CRM_197_). This process is time consuming, requires several rounds of purification to remove toxic chemicals and by products, and can often result in batch to batch variation [17].

An increase in the understanding of bacterial protein glycosylation has led to development of novel ways to couple protein and polysaccharide (reviewed in [18]). The approach, often referred to as protein glycan coupling technology (PGCT) allows production of protein polysaccharide conjugate vaccines in *Escherichia coli* [19]. This technology utilises an oligosaccharyltransferase enzyme, PglB, from the general protein glycosylation locus (Pgl) of *Campylobacter jejuni* [19]. This locus encodes the genes required for the production of a *C. jejuni* heptasaccharide along with PglB, which is required for coupling of the heptasaccharide to a carrier protein. PglB couples over sixty *C. jejuni* proteins to this heptasaccharide [20]. These proteins contain an amino acid acceptor sequence, which is recognised by PglB [21]. The consensus, or glycotag, sequence can be engineered into any protein carrier, allowing recognition by PglB [21]. The glycan specificity of *C. jejuni* PglB has been well characterised, using this knowledge researchers have been able to couple a number of different polysaccharides to chosen carrier proteins using PglB [21], [22], [23]. In simple terms, PGCT can be divided into three procedures. In the first stage, genes encoding the target glycan are faithfully cloned and expressed in *E. coli* on a suitable plasmid. In the second stage, the target carrier protein containing the appropriate consensus sequon and purification tag are cloned into a suitable plasmid, and targeted to the periplasm. Finally, the coupling enzyme, CjPglB, recognises the initial sugar on the glycan and transfers it to the carrier protein. The plasmids are introduced into an appropriate *E. coli* host strain to produce an inexhaustible supply of recombinant glycoprotein that can be readily purified [19].

There are a number of vaccines that have been produced using this technology that show excellent promise in both animal models and in clinical trials [24], [25], [26], [27]. Vaccines produced using PGCT will reduce vaccine costs, speed up the production process, and negate the need for growth of large volumes of pathogenic bacteria. Other benefits of using this technology include the ability to readily change the carrier protein, and to add further compatible polysaccharide types. Further, this technology could allow rapid addition of polysaccharides to vaccine preparations to protect against emerging serotypes. Vaccines produced in this manner could also be tailored to specific geographical regions, by protecting against the most prevalent serotypes. To date pneumococcal polysaccharides of type 4, 5, 8 and 12F have been expressed in *E. coli* [28], the first stage of PGCT.

In the current study, we provide the first evidence that recombinant protein polysaccharide conjugate vaccines can be produced in *E. coli* and protect against pneumococcal invasive disease in a murine infection model.

## 2 Materials and methods

### 2.1 Bacterial strains and plasmids

*Escherichia coli* strains were grown in modified super optimal broth, SSOB (Tryptone 2%, Yeast extract 0.5%, NaCl 10 mM, KCl 2.5 mM, MgCl_2_ 10 mM, MgSO_4_ 10 mM) at 28 °C, with shaking. Antibiotics were added as necessary for plasmid maintenance: tetracycline 20 µg ml^−1^; ampicillin 100 µg ml^−1^; chloramphenicol 30 µg ml^−1^. A table of strains and plasmids used in this study can be found in the supplementary information (Table S1).

*Streptococcus pneumoniae* strain (TIGR4) was cultured on BHI agar with 5% horse blood, or statically in BHI broth, in an atmosphere containing 5% CO_2_.

### 2.2 Vaccine production

Recombinant serotype 4 polysaccharide was produced in *E. coli*, as previously described [28]. Conjugation to AcrA was carried out using protein glycan coupling technology [25]. *E. coli* cultures were grown for 16 h. These starter cultures were used to inoculate 2 L of SSOB to an OD600 of 0.03 and incubated with shaking at 28 °C. Once OD600 had reached 0.4–0.6, expression of PglB was induced with the addition of 1 mM IPTG. MnCl_2_ was also added to a final concentration of 4 mM. After 20 h growth at 28 °C cells were pelleted by centrifugation at 5400*g* for 30 min at 4 °C. Pellets were resuspended in lysis buffer (50 mM NaH_2_PO_4_, 0.3 M NaCl and 10 mM imidazole, pH 7.5) with 1 mg/ml lysozyme, and lysed using a FastPrep instrument (MP Biomedicals) with lysing matrix B. Supernatant was treated with 250 units benzonase for 10 min. Insoluble debris was removed by centrifugation at 7800*g* for 60 min at 4 °C and the supernatant passed through a 0.2 µm filter. The protein/polysaccharide conjugate labeled with a polyhistidine affinity tag was purified using HisTrap columns (GE Healthcare) using an imidazole gradient of 20–300 mM on an AKTA protein purification system (GE Healthcare).

### 2.3 SDS-PAGE and immuno blot analysis

To verify glycoconjugate production and to select AKTA fractions for pooling, samples were subject to SDS-PAGE followed by coomassie staining or immunoblot. Rabbit anti-serotype 4 capsule antibody from the Statens Serum Institut, (SSI) Denmark was used at a dilution of 1:1000 and mouse anti-His monoclonal antibody (Abcam, UK) was used at a dilution of 1:10,000 to detect recombinant serotype 4 capsule and His-tagged AcrA respectively. HR6 antiserum was used to detect the *Campylobacter* heptasaccharide (S. Amber and M. Aebi, unpublished data). Secondary goat anti-rabbit IgG IRDye 800 and goat anti-mouse IgG IRDye 680 conjugates were used at a dilution of 1: 10 000. Fluorescent signal was detected using an Odyssey LI-COR detection system (LI-COR Biosciences UK Ltd.).

### 2.4 Protein and polysaccharide (PS) quantification in vaccine preparations

Selected AKTA fractions were concentrated using Vivaspin protein concentrator spin columns with 10 KDa MWCO (GE Healthcare) and protein was quantified using a Qubit protein assay (Thermo Fisher Scientific). Levels of Type 4 polysaccharide in vaccine preparations was quantified by ELISA using type 4 antiserum and a standard curve generated using purified type 4 polysaccharide (SSI, Denmark).

### 2.5 Vaccination

All *in vivo* experiments were carried out in accordance with the UK Animal Scientific Procedures Act (1986). Mice used in this study were 6–8 week old outbred female MF1 (Harlan, UK). Mice had food and water ad libitum, were kept at a constant room temperature of 20–22 °C, with a 12 h light/dark cycle. For immunization, each mouse received three subcutaneous injections with an interval of two weeks between each. All vaccines were made up in phosphate buffered saline (PBS). For the positive controls mice were vaccinated with pneumococcal 13-valent conjugate vaccine (PCV) Prevnar (Pfizer). Two dosing regimes were used for the Prevnar control groups: PCV13 high vaccination contained 0.5 μg of type 4 PS/dose, while PCV13 low contained 0.0001 μg type 4 PS/dose. The AcrA-SP4 conjugate vaccine contained 0.0001 μg type 4 PS/dose. The AcrA alone and AcrA-Pgl vaccines were normalised to contain the same amount of AcrA to that in the AcrA-SP4 preparation. This was done by western blotting using an anti-His tag antibody (Abcam, UK). Alhydrogel was added to the experimental vaccine preparations to the same level as that found in the PCV13 high dose (Type 4 PS only controls, AcrA only, AcrA-Pgl, AcrA-SP4). Sham vaccination consisted of PBS with Alhydrogel.

### 2.6 Intranasal infection model

Four weeks after the last vaccination mice were challenged with *S. pneumoniae* serotype 4 strain, TIGR4. Mice were inoculated intranasally with 5 × 10^6^ colony forming units (CFU) in 50 μl PBS. All mice were monitored for symptoms and were sacrificed at a designated clinical endpoint point. Organs were removed (lungs, liver, spleen, brain), blood taken and viable counts performed using the Miles and Misra method [29]. Mice that did not reach the clinical endpoint were sacrificed at 7 days post infection and processed in the same way. Blood samples from tail veins, body weight and clinical scores were also taken throughout the study. Graphical representation and statistical analysis was performed in Prism version 4.0b (GraphPad Software), using a non-parametric Mann-Whitney two sample rank test; significance P < 0.05. Survival of mice receiving different vaccine preparations was compared using a Kaplan-Meier survival curve and analysed using a Log-rank Test (P < 0.05).

### 2.7 Preparation of Luminex beads

Protein (AcrA) and polysaccharide (type 4 polysaccharide) were coupled to carboxylate microspheres specific for use in the Luminex multiplex machine as described previously [30], [31]. Briefly, type 4 pneumococcal polysaccharide (SSI 5 mg/ml) was coupled to poly-L-lysine (PLL) using cyanuric chloride. Type 4 PS coupled to PLL was then purified using a G25 PD-10 Sephadex column (GE healthcare). Type 4 PS-PLL was then coupled to carboxylated microspheres (Bio-Plex COOH bead 11, BIO-RAD, UK). AcrA was coupled to a different bead set (Bio-Plex COOH bead 47) at a concentration of 50 μg/ml and did not require prior coupling to PLL. Coupling of beads to antigens was performed using standard methods. Briefly, beads were activated with 5 mg/ml EDC (1-ethyl-3-(3-dimethylaminopropyl)carbodiimide hydrochloride) solution and 5 mg/ml NHS (*N*-hydroxysuccinimide) solution. Bead sets were washed with PBS and incubated with their individual antigen. After incubation, beads were washed in PBS and resuspended in 300 μl PBS 0.1% BSA, 0.05% sodium azide and kept at 4 °C in the dark until used.

### 2.8 Luminex assay antibody quantification

Assays were adapted from [31]. Briefly, sera taken from individual vaccinated mice prior to bacterial challenge were assessed for antibodies against AcrA and type 4 polysaccharide. Serum was diluted 1/100 in sample buffer (PBS 0.05% tween 20, 1% BSA, 5 μg/ml CWPS, 5 μg/ml 22F PS). A standard curve was created from serial 10 fold dilutions of a human standard serum (0 0 7). This serum has known anti-pneumococcal PS antibody titres and allowed extrapolation of anti-type 4 PS antibody levels in mouse sera from the human serum standard curve. For AcrA, antibody levels are based on the mean fluorescent intensity values and are relative quantifications of anti-AcrA antibodies between the different vaccinated groups.

Assays were run in 96-well filter plates (Millipore, UK) with 2500 beads/antigen in each well. To each well 25 μl of diluted sera was added. A type 4 antiserum and a PCV13 mouse control sera were run as internal controls for anti-type 4 PS antibodies for each assay. An in house anti-AcrA antibody was used as an internal control for AcrA samples. Beads and sera were incubated at room temperature for 1 h with shaking at 500 rpm. Beads were washed with PBS 0.05% tween 20 then incubated with a 1/200 dilution of anti-human, anti-rabbit or anti-mouse IgG phycoerythrin (PE) conjugate antibody (Southern Biotech, UK) for 30 min, shaking at 500 rpm. Beads were washed 2× as above and resuspended in 125 μl PBS 0.05% tween 20. Data was acquired on a Luminex-100 instrument (BIO-RAD, UK). Data analysis was performed on Bio-Plex manager 4.1.1 software, which created the standard curve from the 007 human sera. From this anti-type 4 PS, antibody levels in mouse sera were extrapolated. Graphical representation was performed in Prism version 4.0b (GraphPad Software) showing antibody titres in the different vaccinated groups. Statistical analysis was performed using a Kruskal-Wallis one-way ANOVA with a Dunn’s multiple comparison test (P < 0.05).

### 2.9 Opsonophagocytic killing assay (OPKA)

HL60 cells were maintained in RPMI medium 1640 GlutaMAX (Thermofisher, UK) supplemented with 20% fetal calf serum, 1× penicillin streptomycin and 2 mM L-glutamine. Differentiation was performed using a cell density of 4 × 10^5^ cells/ml in 0.8% Dimethylformamide. Cells were differentiated for 5 days and then used in the OPKA. Cells were washed in HBSS−/+ Ca^2+^/Mg^2+^ and resuspended at the desired concentration in opsonisation buffer (OPB buffer) (1 ml 10× HBSS+ Ca^2+^/Mg^2+^,1 ml gelatin, 530 μl Fetal bovine serum, 8 ml H_2_O).

OPKAs were performed on serum samples taken from vaccinated mice. Serum was heat inactivated for 30 min at 56 °C. Serial two-fold dilutions of sera were performed in PBS with a starting dilution of 1:2. TIGR4 was added to each well at a concentration of 2.5 × 10^3^ cfu and incubated for 30 min at 4 °C. Bacteria were pelleted by centrifugation and 15% baby rabbit complement was added, followed by 5 × 10^5^ differentiated HL60 cells. Following incubation at 37 °C in 5% C0_2_ for 40 min, the contents of each well were diluted 1/10 in PBS, and the dilution and neat samples plated onto BAB plates containing 5% horse blood. Plates were incubated overnight at 37 °C in 5% CO_2_. Colonies were counted and percentage killing calculated in comparison to the bacteria, complement and cells only control (no sera added). Type 4 antiserum (SSI) and an in house PCV13 mouse control serum were used as internal controls. Graphical representation was performed in Prism version 4.0b (GraphPad Software) showing percentage killing of TIGR4 with sera from vaccinated mice relative to the bacteria, complement and HL60 cells only control.

The datasets generated during and/or analysed during the current study are available from the corresponding author on reasonable request.

## 3 Results

### 3.1 Coupling of serotype 4 pneumococcal polysaccharide to AcrA

**Fig. 1:**
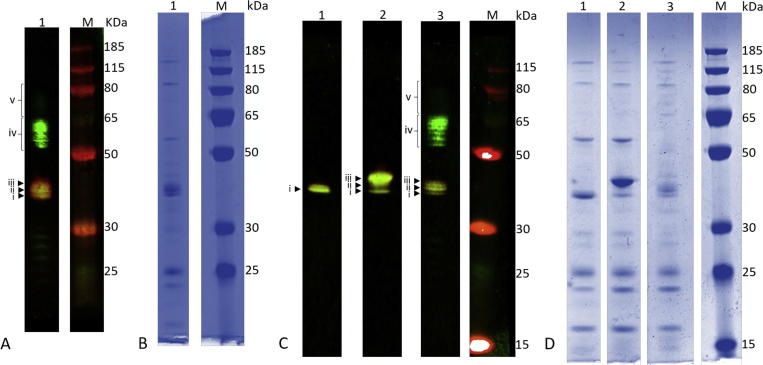
Recombinant glycoconjugate vaccine preparations produced in *E. coli*. 0.5 µg protein separated on SDS-PAGE gel for batch 1 (A/B) and batch 2 (C/D), A/C – Immunoblot with anti-His tag antibody (red) and anti- SP4 capsule antibody (green). SP4 antiserum cross reacts with AcrA. B/D – Coomassie stained gel. A/B. Lane 1: AcrA conjugated to recombinant SP4 polysaccharide. C/D Lane 1: AcrA only; lane 2: AcrA conjugated to *C. jejuni* heptasaccharide; lane 3: AcrA conjugated to recombinant SP4 polysaccharide. M: molecular weight marker PageRuler Plus. i = unglycosylated AcrA, ii = AcrA glycosylated with single glycan unit at one glycosylation site, iii = AcrA glycosylated with single glycan unit at both glycosylation sites, iv = AcrA glycosylated with polymerized SP4 at one glycosylation site, v = AcrA glycosylated with polymerized SP4 at both glycosylation sites. Images have been cropped for clarity and ease of labelling. Each sub figure contains lanes cropped from the same gel. Separate, uncropped figures for each of the fluorescence channels are presented in supplementary data (Fig. S2 and S3). (For interpretation of the references to colour in this figure legend, the reader is referred to the web version of this article.)

The oligosaccharyltransferase, CjPglB, was used to transfer recombinantly expressed *S. pneumoniae* serotype 4 capsular polysaccharide to AcrA. CjPglB covalently attaches glycans to asparagine residues, within a conserved motif, via an N-glycosidic bond [32], [21]. The *pglB* gene was introduced onto the chromosome of *E. coli* wild type strain W3110, to form strain W311B (Abouelhadid et al. manuscript in preparation). Previous work has shown that *S. pneumoniae* serotype 4 capsular polysaccharide can be recombinantly expressed in *E. coli* using the plasmid, pB-4 [28]. AcrA is a protein that forms part of a multidrug efflux pump in *C. jejuni* [33], and is known to be glycosylated with a heptasaccharide via PglB *in vivo* [19]. AcrA has also been used as a glycan carrier in a conjugate vaccine produced against brucellosis [34]. In this study, *E. coli* strain W311B was transformed with plasmids pB-4 (carrying the serotype 4 capsule locus) and pWA2 (carrying *acrA*) to generate a glycoconjugate vaccine consisting of AcrA coupled to type 4 polysaccharide. Control strains were also generated by transformation of W311B: with pWA2 only, to express AcrA; or in combination with pPgl (pACYC carrying the whole *pgl* locus from *C. jejuni* with *pglB* mutated) to conjugate the *C. jejuni* heptasaccharide to AcrA. Following overnight induction with IPTG, cells were lysed and the AcrA protein was purified by affinity chromatography. 0.5 µg of protein purified from each recombinant strain was analysed by SDS-PAGE and immunoblotting (Fig. 1). When AcrA alone is expressed, a single band of 40 KDa is visualized with the anti-His tag antibody (Lane 1 in Fig. 1C). AcrA conjugated to the *C. jejuni* heptasaccharide has three his-reactive bands, with the most abundant band being the highest, corresponding to AcrA glycosylated at both sites with the *C. jejuni* heptasaccharide, which does not form a polymer (Lane 2 in Fig. 1C). Yield of conjugate vaccine was low (Table 1: 201 μg and 180 μg per g wet-weight of *E. coli*, for AcrA-SP4 P1 and P2 respectively) and therefore two separate batches of vaccine were purified for mouse vaccination and protection studies (Fig. 1: Panel A1 and C3). In the AcrA-SP4 samples, three bands react with the anti-His tag antibody. One of these three bands has the same molecular weight as AcrA and is therefore predicted to be non-glycosylated AcrA. The two higher molecular weight bands represent AcrA glycosylated at one and two glycosylation sites. A further group of bands at higher molecular weights can be seen above the protein, representing polymer chains attached at one and two sites.

**Table 1:** Comparison of SP4-AcrA P1 and P2.

	SP4-AcrA P1	SP4-AcrA P2
Yield per g of cells	201 μg	180 μg
% glycosylated AcrA	80%	84%
AcrA attached to polymer	3.6%	2.8%
AcrA per dose of vaccine	26.8 μg	15.9 μg

### *3.2* *In vivo* vaccine efficacy

To assess the protective efficacy of the recombinant glycoconjugate vaccine containing type 4 polysaccharide coupled to AcrA (AcrA-SP4), mice were vaccinated with conjugates followed by challenge with *S. pneumoniae* strain TIGR4. Type 4 polysaccharide was chosen to test the conjugation approach because the unconjugated polysaccharide is not immunogenic in mice [35]. This allowed us to test the immunogenicity of the conjugate alone as no protection would be observed from any free polysaccharide present in the vaccine preparations [35].

The amount of type 4 PS in the vaccine preparation was measured using ELISA against a type 4 PS standard serum from SSI (data not shown). The quantity of AcrA in the preparations was measured by western blot using a HIS-tag antibody, and the amount of AcrA normalised between the AcrA containing preparations (data not shown). PCV13 was used as a positive control and the amount of type 4 polysaccharide in PCV13 was matched to that in the AcrA-SP4 conjugate (0.0001 μg). This was designated as Prevnar low dose (PLD) due to the relatively low amount of type 4 polysaccharide in AcrA-SP4. A higher dose of PCV13 (PHD) was also given as a positive control, as our previous studies showed this was protective in our mouse model of infection (containing 0.5 μg type 4 polysaccharide/dose).

Two separate batches of the experimental conjugate were tested.

MF1 outbred mice were vaccinated with three doses of: AcrA-SP4, Prevnar13 (PHD/PHL) or one of the control groups (unconjugated type 4 PS (high/low dose), AcrA alone, AcrA coupled to its native *C. jejuni* heptasaccharide (AcrA-Pgl) or sham vaccinated). Four weeks post the last vaccination mice were challenged via the intranasal route with a serotype 4 *S. pneumoniae* strain (TIGR4). Disease progression was assessed in all vaccinated groups.

Initial experiments used three mice to test the first vaccine preparation, referred to as AcrA-SP4 P1. Small groups of mice were used due to amounts of conjugate required for the vaccination schedule. Further experiments were performed using a second batch of conjugate vaccine, referred to as AcrA-SP4 P2 (n = 4). Data is presented separately for the two groups due to the differing results observed for the two preparations.

**Fig. 2:**
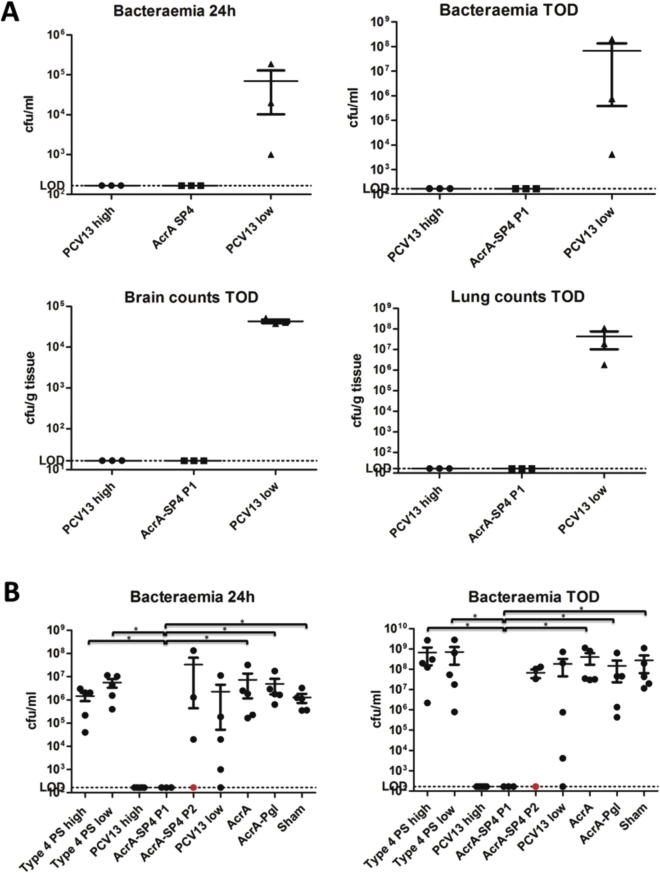
Bacterial counts in Blood, Brain and Lungs. Fig. 2A shows Levels of bacteria in the blood, lungs and brain of vaccinated mice (preparation 1) post challenge with TIGR4. Bacteria in the blood is shown at 24 h post challenge (taken via tail vein bleed) and at time of death (TOD, taken via cardiac puncture under terminal anesthesia). No statistical analysis was performed on data, n = 3 mice/group. Fig. 2B shows levels of bacteria in the blood of vaccinated mice (preparation 1 and 2) at 24 h post challenge with TIGR4 (tail vein bleed) and at the TOD. Red circular symbol represents data from the mouse that was vaccinated with AcrA-SP4 P2 and survived challenge. Statistical analysis was performed in GraphPad Prism using a non-parametric Mann-Whitney two sample rank test, significance *P < 0.05, n = 3–5 mice/group. Each dot represents counts from a single animal.

In the initial experiment, using Preparation 1 of the AcrA-SP4 vaccine, three mice were vaccinated for each group. All mice in the PHD vaccinated group survived the infection, whereas all mice in the PLD group succumbed. The experimental AcrA-SP4 conjugate vaccine conferred 100% protection. Analysis of bacterial counts in the lungs, brain and blood showed no bacteria present above the limit of detection in the organs of mice vaccinated with PHD or AcrA-SP4 P1 (Fig. 2A). Due to the low numbers of mice, statistical analysis was not performed.

Next we attempted to repeat these experiments with a second batch of AcrA-SP4 conjugate (AcrA-SP4 P2). However, in this series of experiments only one mouse from the group of 4 vaccinated, survived bacterial challenge. The surviving mouse showed no bacterial counts in the blood at 24 h and at the time of death (Fig. 2B). Whereas, the three mice that succumbed to infection all had bacteria in the blood at 24 h post infection, similar to that observed in the control groups. The PHD positive control group was protected from infection and showed significantly lower (P < 0.05) bacterial counts in the blood compared to all control groups (except AcrA-SP4, analysis was performed on all AcrA-SP4 vaccinated mice together (n = 7)), data not shown on graph. Mice vaccinated with AcrA-SP4 P1 had significantly lower bacterial counts in the blood at both time points compared to the T4PS high/low, AcrA, AcrA-Pgl and sham vaccinated control groups. This was not observed with mice vaccinated with AcrA-SP4 P2.

**Fig. 3:**
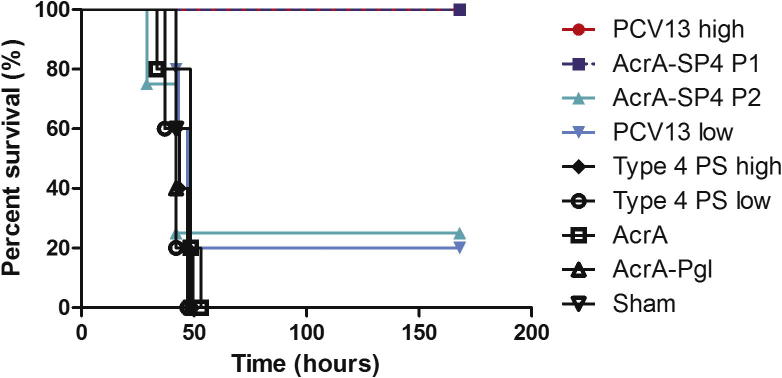
Survival of vaccinated mice post intranasal challenge with TIGR4. This figure shows survival of vaccinated mice following challenge with TIGR4. Mice were followed for disease progression, once clinical symptoms reached a designated endpoint mice were sacrificed. Experimental endpoint was 7 days post challenge (168 h) and surviving mice were sacrificed at this time point. n = 5 mice/group bar AcrA-SP4 P1 where n = 3 and AcrA-SP4 P2 where n = 4. Statistical analysis was performed in GraphPad Prism using a Log-rank Test (P < 0.05).

Survival curves were used to compare the two preparations (Fig. 3). Mice vaccinated with AcrA-SP4 P1 showed a 100% survival rate (n = 3), whereas mice vaccinated with AcrA-SP4 P2 only had a 25% survival rate (n = 4). Despite a trend towards a difference there was no significant difference between survival rates with mice vaccinated with AcrA-SP4 P1 and P2 (P 0.0798). No significant difference was observed between PHD and AcrA-SP4 P1 vaccinated mice. Further, all control groups (sham, T4PS high/low, AcrA and AcrA-Pgl) showed a significant reduction in survival compared to mice vaccinated with AcrA-SP4 P1. No significant difference in survival was observed between control groups (sham, T4PS high/low, AcrA and AcrA-Pgl) and mice vaccinated with AcrA-SP4 P2.

Using Western blot analysis, the two AcrA-SP4 vaccine preparations were compared using fluorescent intensity measurements (supplementary Fig. 1). As the SSI SP4 antiserum cross-reacts with AcrA all measurements were confined to the anti-his channel. This analysis revealed a higher percentage glycosylation for AcrA-SP4 P2 (84% Vs 80%) but a lower percentage of AcrA coupled to polymers of SP4 (2.8% Vs 3.6%). In addition, by normalizing to glycan content of the preparations, the protein content per dose of the P1 was 26.8 μg vs 15.9 μg in P2 (Table 1 and supplementary Fig. 1).

In an attempt to explain the variation in levels of protection of the two vaccine preparations, we examined the antibody levels and functionality of the antibodies generated by the two vaccines.

### 3.3 Vaccine induced antibody responses

**Fig. 4:**
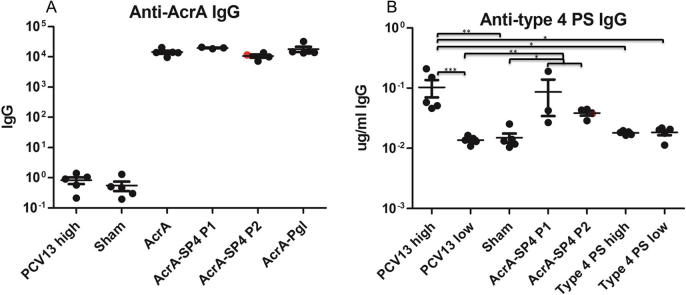
Antibody responces in vaccinated mice. A shows the IgG antibody responces to the carrier protein AcrA in vaccinated mice, measured using a luminex assay. B shows the IgG antibody responces to type 4 polysaccharide in vaccinated mice, measured using a Luminex assay. Antibody responces were measured in sera taken pre challenge with TIGR4. Each dot represent antibody levels in a single animal. Red circular symbol indicates the antibody levels in the mouse that was vaccinated with AcrA-SP4 P2 and survived challenge. Statistical analysis was performed in GraphPad Prism using a Kruskal-Wallis one way ANOVA with a Dunn’s multiple comparison test, *P < 0.05, **P < 0.01, ***P < 0.001. (For interpretation of the references to colour in this figure legend, the reader is referred to the web version of this article.)

A Luminex bead based assay was used to evaluate the amount of antibody produced against type 4 PS and the carrier protein AcrA, in serum from vaccinated mice (Fig. 4). Sera used were obtained from tail vein bleeds taken directly before challenge with TIGR4. Type 4 PS levels are given in μg/ml as samples were compared to a standard human serum with known levels of anti-type 4 PS antibody (μg/ml) [36]. AcrA levels are presented as relative amounts of IgG between the different samples, as no serum standard with known levels of AcrA antibody was available.

Anti-AcrA antibodies were detected in all serum samples taken from mice that were vaccinated with AcrA conjugates (AcrA-SP4/AcrA-Pgl) or AcrA alone (Fig. 4A). The AcrA-SP4 vaccinated mice antibody responses have been split into those that were vaccinated with the first (AcrA-SP4 P1 n = 3) and second preparation (AcrA-SP4 P2 n = 4). The antibody titer from the mouse that was vaccinated with the second preparation and survived is shown by a red circular symbol. There was no significant difference in the levels of anti-AcrA antibodies in AcrA vaccinated groups, suggesting this is not the cause of the difference in survival.

Anti-type 4 PS antibody levels in vaccinated groups are shown in Fig. 4B. The AcrA-SP4 vaccinated mice antibody responses have been split into those that were vaccinated with the first (AcrA-SP4 P1 n = 3) and second preparation (AcrA-SP4 P2 n = 4). Anti-type 4 PS antibody levels were detectable in the PHD vaccinated mice and AcrA-SP4 vaccinated mice. There was no significant difference in antibody levels between the PHD group and the two AcrA-SP4 groups (analysis was performed on all AcrA-SP4 vaccinated mice together (n = 7)). There were significantly lower levels of anti-type 4 PS antibodies in the PLD vaccinated group compared to PHD high (P = <0.001) and the AcrA-SP4 vaccinated group (n = 7, P = <0.01), suggesting the lower PCV13 dose is not sufficient to produce detectable levels of anti-type 4 PS antibodies in our model. When splitting the AcrA-SP4 vaccinated mice into the two preparations there was no significant difference in the anti-type 4 PS levels between the AcrA-SP4 mice vaccinated with the first or second vaccine preparation. This suggests that the mice that succumbed to infection did have anti-type 4 PS antibodies, but these were not sufficient to clear infection. We know from previous literature [35] that unconjugated type 4 PS is not immunogenic in mice, and this is confirmed in our model as no anti-type 4 PS antibodies were detected in the polysaccharide only control groups.

WHO has assigned a protective IgG serotype specific antibody level of 0.35 μg/ml as measured by ELISA [37]. However, it has been shown that this does not necessarily correlate with protection; OPKA is seen to be a better measure of true protection [38], [39]. There was no significant difference in the total anti-type 4 PS IgG antibody levels in mice vaccinated with the first or second AcrA-SP4 vaccine preparation. Therefore, the ability of the antibodies to opsonise TIGR4 was tested using an OPKA. Dilutions of sera from single vaccinated mice were incubated with TIGR4, differentiated HL-60 cells, and a complement source. Percentage killing of TIGR4, when incubated with sera from vaccinated mice, was compared to samples that contained no sera (bacteria, cells and complement only control). In our assay, all sera that resulted in bacterial killing above 50% is classed as protective. This assay was then used to test functional antibodies in the sera from mice vaccinated with AcrA-SP4.

**Fig. 5:**
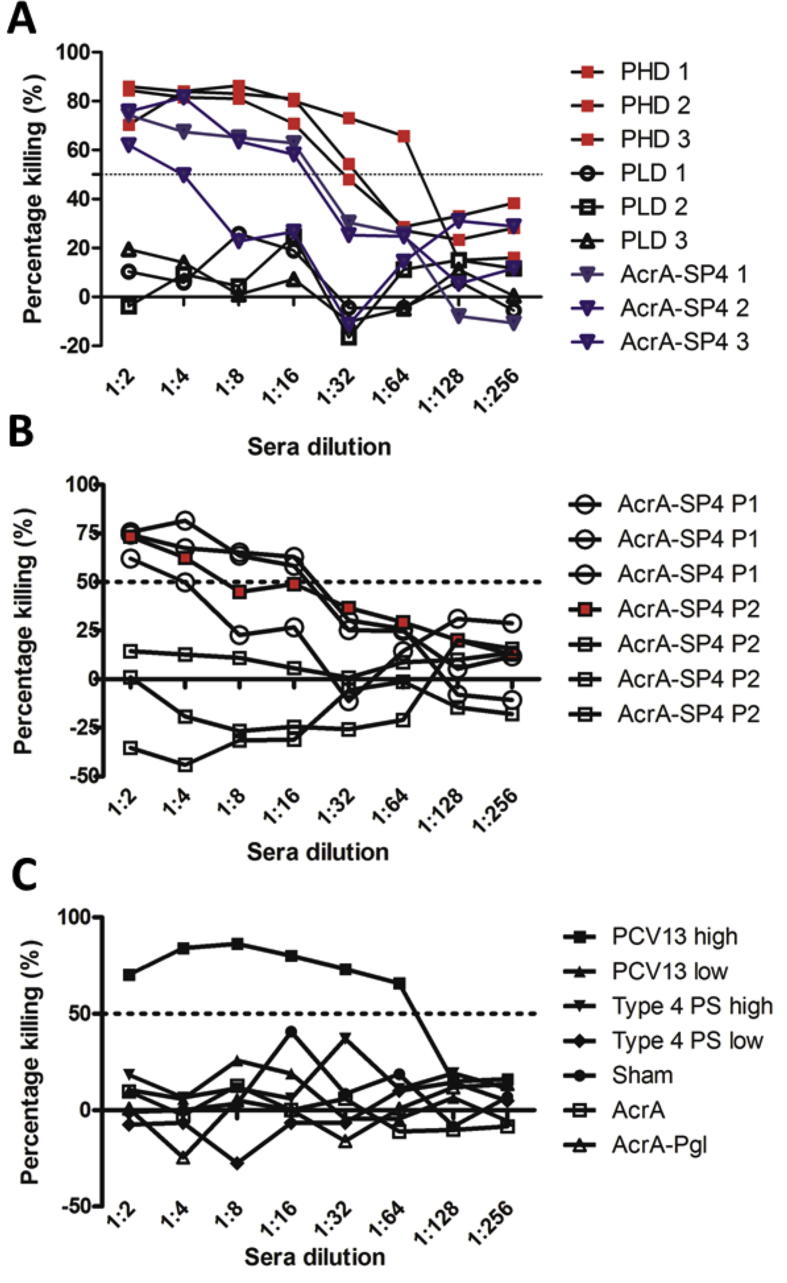
Functional antibody levels in vaccinated mice. (A) shows functional antibodies in sera from mice vaccinated with AcrA-SP4 P1 (blue), PHD (red) and PLD (black). Each line represents sera from a single mouse. Percentage killing of TIGR4 is shown at dilutions of sera starting at 1:2 dilution. (B) shows functional antibodies in sera from all seven mice vaccinated with AcrA-SP4 P1 and AcrA-SP4 P2. Each line represents sera from a single mouse with the circular symbol representing the mice that survived TIGR4 intranasal infection, and open square those that succumbed to infection. Red filled square symbol indicated the functional antibody levels in the mouse that survived from the second vaccine preparation group. (C) shows functional antibodies in sera from a single mouse from each of the control groups. Percentage killing of TIGR4 is shown at dilutions of sera starting at 1:2 dilution. (For interpretation of the references to colour in this figure legend, the reader is referred to the web version of this article.)

Initial experiments were performed on sera from mice given the first vaccine preparation. Sera samples taken from vaccinated mice were tested individually. Samples were run alongside a standard type 4 PS antisera (SSI) to assess any intra-assay variability (data not shown). This assay confirmed whether antibodies produced from vaccination of mice were able to opsonise and kill TIGR4 *in vitro*, which has been shown to correlate with protection [40]. Data in Fig. 5A shows the percentage killing of TIGR4 from the three vaccinated mice with AcrA-SP4 P1, PHD and PLD. All mice vaccinated with PHD and AcrA-SP4 P1 showed opsonic antibodies above the 50% killing cut off. The three mice vaccinated with the PLD did not show this and all mice in this group succumbed to infection.

This assay was then used to test the opsonic antibody levels in the mice vaccinated with AcrA-SP4 P2, and compared to the AcrA-SP4 P1 data (Fig. 5B). One serum sample taken from a mouse in each of the control groups was also tested (Fig. 5C). From our OPKA data, we observe functional antibodies (killing above 50%) in all sera samples from mice that survived intranasal challenge with TIGR4. In the seven mice that received AcrA-SP4, functional antibodies were observed in the four mice that survived; this was not the case for the three mice that succumbed to infection. Sera from mice that were sacrificed due to clinical disease symptoms did not reach killing above the 50% threshold. Data from the mouse that was vaccinated with the AcrA-SP4 P2 but survived challenge is shown as a red square symbol (Fig. 5B). We observed high levels of killing in the sera from one of the PCV13 low vaccinated mice, which survived challenge (data not shown). For the other control groups, percentage killing did not reach above 50%; again, this correlated to the survival status of the mouse. There is therefore a strong correlation between the ability of the antisera to promote opsonophagocytosis *in vitro* and the ability to protect from infection *in vivo*.

## 4 Discussion

We report the first demonstration of the use of protein glycan coupling technology (PGCT) to produce a recombinant pneumococcal conjugate vaccine candidate. We have provided the first proof of principle that a recombinant pneumococcal conjugate vaccine can be protective in a mouse model of infection, and can produce functional opsonic antibodies. Production of these opsonic anti-capsular antibodies correlates with the ability to protect from lethal infection in a mouse model of disease.

Glycoconjugate vaccines are used worldwide to prevent disease in children caused by encapsulated bacteria. In the UK, conjugate vaccines are given in the routine childhood vaccination schedule to prevent disease from three important pathogens: *H. influenza type B*, *N. meningitides*, and *S. pneumoniae*. These vaccines are all composed of type specific polysaccharide coupled to a carrier protein, normally diphtheria toxoid or tetanus toxoid. Despite large reductions in disease caused by these pathogens since conjugate vaccine introduction, these pathogens still remain the three biggest contributors to invasive disease in children worldwide [1], [41], [42]. Conjugate vaccines that protect against pneumococcal infection are known for their safety and efficacy. Producing protective type specific opsonic antibodies [5]. However pneumococcal pneumonia remains one of the biggest killers, resulting in 393,000 deaths of children under 5 in 2015 [43].

This is mainly due to the limited availability of these vaccines in the developing world, due to the large cost [12]. Introduction of conjugate vaccines into a countries vaccine schedules is extremely costly. Many developing countries are only able to afford these vaccines with assistance from The Global Vaccine Alliance (GAVI), which is not sustainable. If eligibility for financial help is lost this leaves a huge financial gap to fill.

PGCT is an alternative approach to making conjugate vaccines. Producing conjugate vaccines in *E. coli* without the need for complex chemical coupling, and growth of large quantities of pathogenic bacteria. Once optimised, this production method has the potential to significantly reduce the cost of conjugate vaccines, making them more readily available globally. This production method will not help with the problem of serotype replacement, but an easier production method may allow more rapid switching of vaccine constituents, to include compatible emerging serotypes into the preparation.

This technology has recently been used for production of a number of novel vaccines. These include vaccines that protect against *Francisella tularensis*, *Burkholderia pseudomellia* and *Staphylococcus aureus* [24], [25], [26]. These all used the CjPglB enzyme to couple O-antigen (gram negative bacteria) or type specific polysaccharide (gram positive bacteria) to a carrier protein of choice, in this instance to exotoxin A from *Pseudomonas aeuriginosa (F. tularensis* and *S. aureus*) or AcrA from *C. jejuni* (*B. pseudomellia*). These glycoconjugate vaccines produced using recombinant DNA approaches all showed at least partial protection in a mouse model of infection, and IgG antibody responses against the vaccine components. This methodology has also been used for production of a glycoconjugate vaccine to prevent disease caused by *Shigella flexneri* 2a [27], coupling the O-antigen to exotoxin A (*P. aeuriginosa*) using PglB. This conjugate vaccine composition produced through chemical coupling has already been shown to be protective and safe in clinical trials [44], however remains expensive to produce.

Using two separately prepared batches of vaccine, we observed a 100% and 25% survival rate of mice vaccinated with AcrA-SP4 compared to 25% of mice vaccinated with the equivalent dose of Prevnar13 (same concentration of type 4 PS – 0.0001 μg). Higher survival rates of 100% were observed with the higher Prevnar13 dose, which is equal to each mouse receiving 100 µl of the neat PCV13 vaccine per dose. Based on the PCV13 vaccine dose, and weight of a 6 week old child, we have estimated a matched dose for a mouse would contain 0.01 µg of type 4 PS/dose. This dosage of 0.01 µg type 4 PS, when used in our infection model, was 100% protective (data not shown). However, due to small amount of AcrA-SP4 conjugate available we were unable to match these doses, and each mouse received 0.0001 µg of type 4 PS per dose.

We believe the amount of AcrA-SP4 conjugate given to each mouse was on the cusp of the amount required to protect against challenge, and different vaccine efficacies observed to the two vaccine preparations is likely due to batch-to-batch variation. Optimisation therefore is required to ensure a uniform batch, stability post purification and the ability to scale up production.

The variation between batches may be due to reduced amounts of coupled protein and polysaccharide present within the second preparation. Total polysaccharide (type 4 PS) was quantified in the vaccine preparations, and this was used to normalise doses between the two experiments. However, this would not tell us if the levels of protein and polysaccharide coupled varied between preparations. To try and evaluate this the two vaccine preparations were coupled to a Luminex assay bead set. The coupling method used was for protein, so only the AcrA in the preparations would bind to the beads. To measure the amount of AcrA coupled to SP4, a secondary type 4 antisera was used (SSI) to quantify the amount of T4PS coupled to the beads, followed by a PE conjugated antibody (data not shown). Fluorescence readings were higher for preparation one, indicating this preparation contained higher amounts of AcrA coupled to T4PS. Type 4 polysaccharide alone is not protective in a mouse model of infection [35]. Therefore, if the level of free polysaccharide was higher in the second preparation this might account for the decrease in protection.

Another possibility for the reduced efficacy in the second preparation could be the composition of repeat unit lengths. It has been shown previously that for *S. pneumoniae* serotype 4 glycoconjugates, a shorter chain lengths (12 repeat units), and a higher ratio of saccharide to protein is required for optimal immunogenicity, than for fully polymerized glycan [35]. Also, for serotype 14 the length of coupled polysaccharide is important for induction of antibodies with high opsonophagocytic activity; higher chain lengths confirm higher opsonophagocytic titres [45]. However, this has not been shown for other serotypes tested [46].

Relative amounts of IgG against AcrA were measured in all vaccinated mice. This carrier protein is not a native pneumococcal protein and therefore provided no protection to mice. This was clearly shown in the AcrA only and AcrA-Pgl vaccinated mice, which all had anti-AcrA IgG antibodies in sera, yet all mice succumbed to infection. This was further validated using our OPKA, as sera from vaccinated mice, in these two groups, were unable to opsonise and kill TIGR4. A benefit of using PGCT, is that alternative protein carriers, that contribute to vaccine efficacy, could be easily incorporated into vaccine preparations. One potential candidate to act as a carrier protein is pneumolysin (Ply), the pneumococcal cholesterol dependent cytolysin. This protein is highly conserved in almost all pneumococcal strains and serotypes [47]. Further, a detoxified version of *ply* (Δ6ply) has been shown to act as an adjuvant, and upon vaccination, results in antibodies produced against itself and any proteins coupled to it [48], [49]. These properties are retained if given mucosally, and would enable the vaccine to be given intranasally [49], a less invasive route than Prevnar13 is currently given (intramuscularly).

Total IgG levels against T4PS in mice vaccinated with AcrA-SP4 did not seem to correlate to survival in our model. When the AcrA-SP4 vaccinated groups were split into those that survived and those that died post challenge with TIGR4, there was no significant difference in antibody levels. For certain serotypes, total IgG levels, as measured by ELISA, have been shown not to correlate with protection, and opsonic antibody activity has been shown to be a more reliable measure of protection [38], [39]. This was also the case in this study, with opsonic antibody activity directly correlating to survival.

In future, it may be informative to study antibody isotypes. Pneumococcal conjugate vaccines have been shown to produce a predominant IgG1 followed by IgG3 antibody response in mice [35]. Response to polysaccharide alone manifest in a predominantly IgG2 and IgM response [35], [50]. Increased levels of IgM or IgG2 in mice vaccinated with the second preparation would have supported the theory of increased free polysaccharide within this preparation.

There are some limitations to overcome before this methodology can compete with traditional chemical coupling. Currently the use of the CjPglB is limited to coupling polysaccharides which have an acetamido group at the C2 position of the reducing end sugar [22]. This only includes a small number of the pneumococcal polysaccharides. Work is underway to overcome these limitations. CjPglB is not only found in *campylobacter* species, and orthologues have also been found in a number of other epsilon proteobacteria [51]*.* This opens up the potential to find glycosyltransferases that have differing or relaxed glycan specificities to that of CjPglB. More recently the crystal structure of PglB from *Campylobacter lari* has been solved giving an insight into the important structural regions required for PglB function [52]. With this knowledge, it is possible to modify PglB to increase transfer efficiency and alter the glycan specificity [53].

## 5 Conclusions

Here we have provided the first proof of principle that a pneumococcal conjugate vaccine, produced by protein glycan coupling technology, is protective in a mouse model of infection, and can produce functional opsonic antibodies. A 57% survival rate of mice vaccinated with the novel conjugate was seen, although there was variation between vaccine preparations. This is a proof of principle study and more work needs to be done to optimise production methodology, to improve polymer length, conjugation efficiency and overall vaccine yield, in order to make a reliable and economical alternative to chemical conjugation methods.

## Competing interests

We have no competing interests.

## Author contributions

J.A.H carried out all animal work and sample processing, Luminex assays and OPKA assays. Further participated in design of the study and drafted the manuscript. E.K produced the vaccine preparations and ran the western blots for vaccine validation. E.K participated in editing the manuscript. S.F helped with the Luminex assays and analysis. S.A. and J.C. developed the genetic tools and generated the chromosomally inserted PglB strain. T.M and B.W conceived of the study and revised the manuscript.

## Funding

This work was supported by a Medical Research Council (MRC) Grant MR/K012053/1 to BW and TJM.
