# Supplementary material for: Production and efficacy of a low-cost recombinant pneumococcal protein polysaccharide conjugate vaccine
Source: Vaccine. 2018 Jun 18;36(26):3809–19. doi: 10.1016/j.vaccine.2018.05.036 (PMC5999350; doi:10.1016/j.vaccine.2018.05.036)
Supplement: Supplementary data 1 [file mmc1.docx]

Table S1: Strains and plasmids used in this study

| Strain/ plasmid | Description | Source/ reference |
| --- | --- | --- |
| *E. coli* W3110 | F- lambda- IN(rrnD-rrnE)1 rph-1 | ^54^ |
| *S. pneumoniae* Serotype 4 strain | Strain 600/62  TIGR4 | SSI, Denmark  ^55^ |
| pB-4 | pBBR1MCS-3 containing a 14095 bp region including *wciI-fnlC*, from *S. pneumoniae* serotype 4, synthesised by Epoch Life Sciences, cloned into KpnI XbaI sites of the MCS. Tet^R^ | ^28^ |
| pWA2 | *C. jejuni* *acrA* soluble with a *pelB* signal sequence and His6-tag, cloned into pBR322. | ^23^ |
| pPgl | pACYCpglB::Km. Pgl locus of *C. jejuni* strain 81116 from pglA-wlaB in pACYC cloning vector. *pglB* interrupted with EZ::Tn transposon system. Cm^R^ Km^R^ | ^56^ |
