# Supplementary material for: Production and efficacy of a low-cost recombinant pneumococcal protein polysaccharide conjugate vaccine
Source: Vaccine. 2018 Jun 18;36(26):3809–19. doi: 10.1016/j.vaccine.2018.05.036 (PMC5999350; doi:10.1016/j.vaccine.2018.05.036)
Supplement: Supplementary data 2 [file mmc2.docx]

Supplementary Information


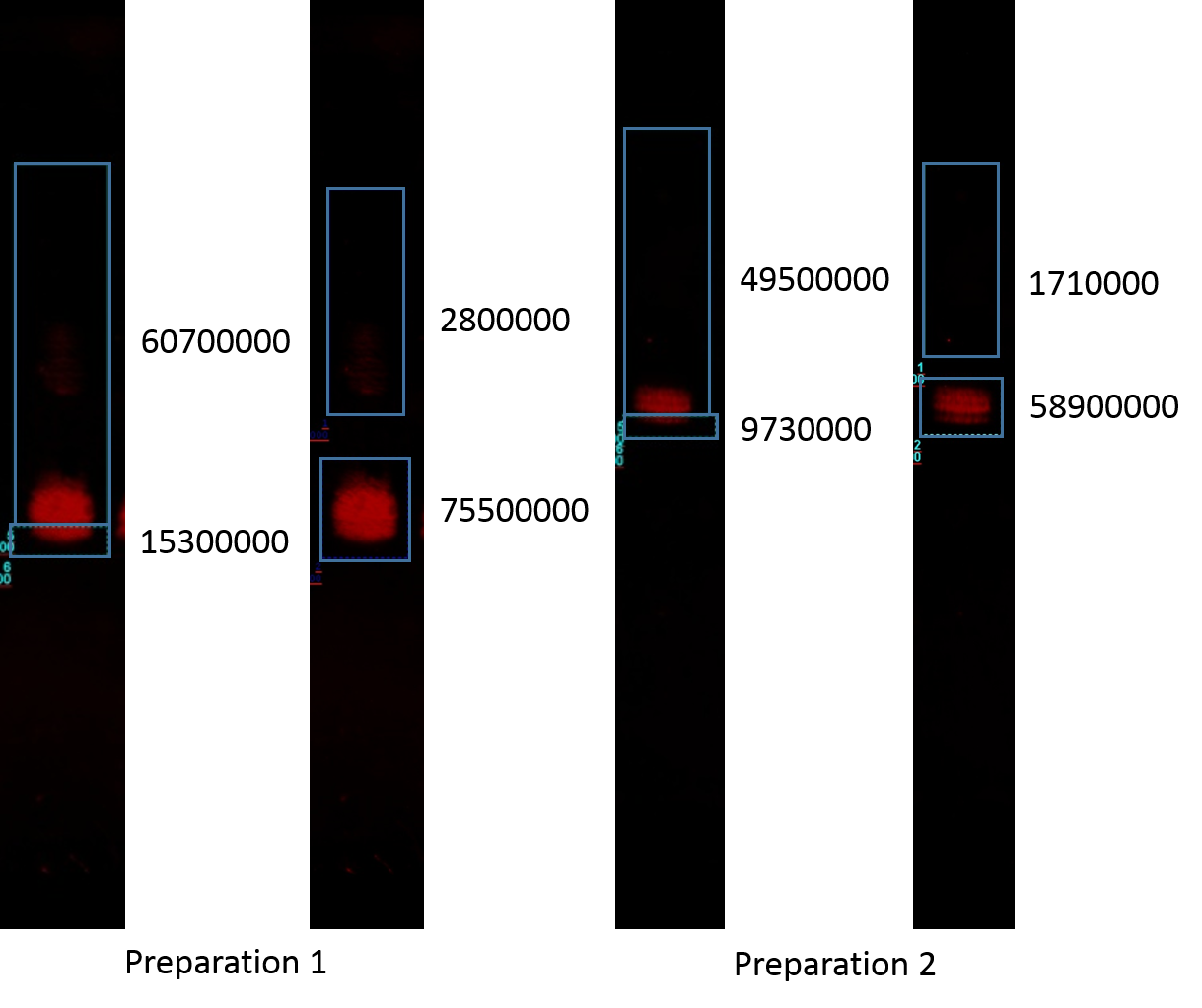


**Supplementary figure 1: Western blot image analysis.** Fluorescence signal intensity was measured using Li-Cor Image Studio software for the 700 channel, corresponding to the anti-His fluorescent secondary antibody and therefore a marker for protein content. Signal within the gated areas is presented as total signal - (Area of the shape x Background). Background is calculated from the median intensity of pixels in a border around the shape.


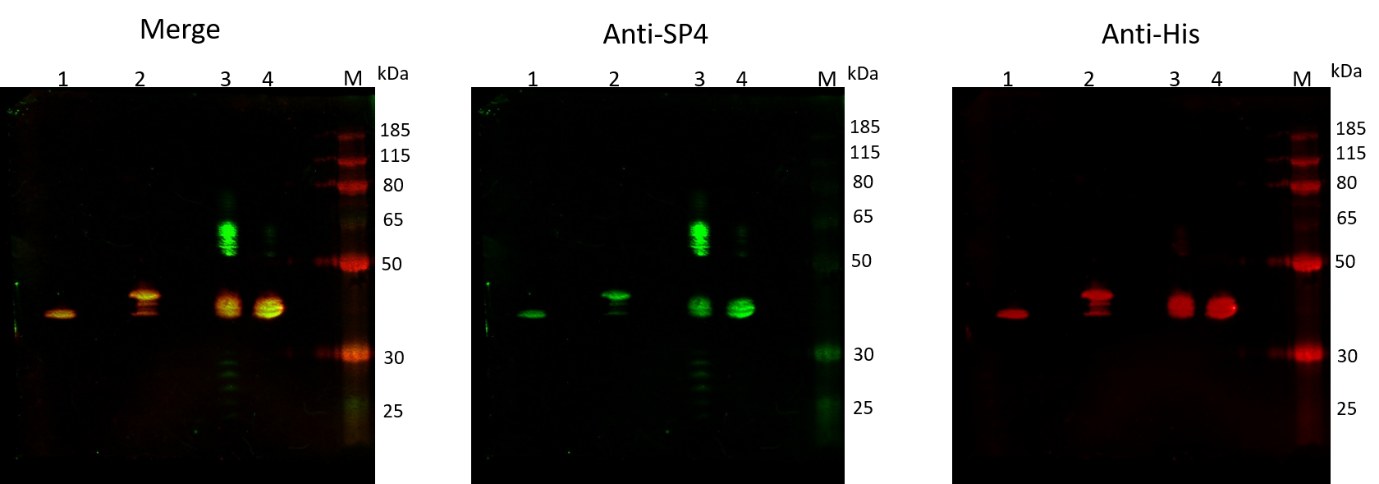


**Supplementary Figure 2: Vaccine preparation 1.** 0.5 µg protein separated on an SDS-PAGE gel. Immunoblot with anti- SP4 capsule antiserum (green – LiCor 800 nm channel) and anti-His tag antibody (red – LiCor 700 nm channel). The same blot was scanned on each fluorescent channel and images presented separately and merged. 1 ml AKTA elution fractions were concentrated through Vivaspin protein concentrator spin columns. Lane 1: AcrA, concentrated AKTA fractions A14-B6. Lane 2: AcrA-Pgl (AcrA conjugated to *C. jejuni* heptasaccharide), concentrated AKTA fractions A13-B6. Lane 3: AcrA-SP4 (AcrA conjugated to recombinant *S. pneumoniae* serotype 4 polysaccharide), concentrated AKTA fractions A12-B2. Lane 4: AcrA-SP4 (AcrA conjugated to recombinant *S. pneumoniae* serotype 4 polysaccharide), concentrated AKTA fractions B3-B6. M: molecular weight marker PageRuler Plus. The serotype 4 antiserum cross-reacts with AcrA. Only sample from lane 3 was used in further experiments.


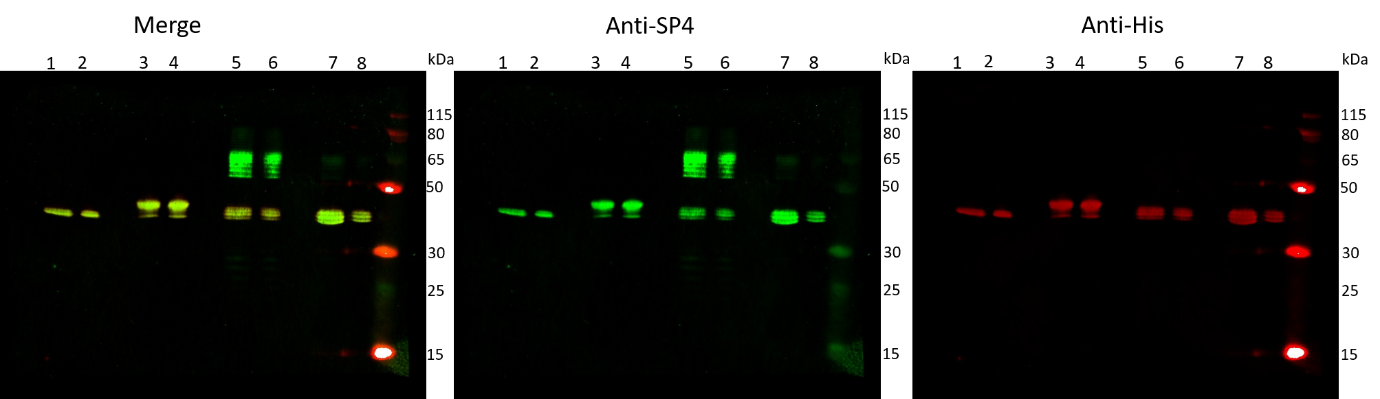


**Supplementary Figure 3: Vaccine preparation 2.** 0.5 µg protein separated on an SDS-PAGE gel. Immunoblot with anti- SP4 capsule antiserum (green – LiCor 800 nm channel) and anti-His tag antibody (red – LiCor 700 nm channel). The same blot was scanned on each fluorescent channel and images presented separately and merged. 1 ml polyhistadine affinity tag purified AKTA elution fractions pooled (lanes 1, 3, 5 and 7) and then concentrated through Vivaspin protein concentrator spin columns (Lanes 2, 4, 6 and 8 - 1/10^th^ sample loaded, quantified as 0.5 µg). Lane 1 and 2: AcrA, AKTA fractions A12-B4. Lane 3 and 4: AcrA-Pgl (AcrA conjugated to *C. jejuni* heptasaccharide), AKTA fractions A12-B5. Lane 5 and 6: AcrA-SP4 (AcrA conjugated to recombinant *S. pneumoniae* serotype 4 polysaccharide), AKTA fractions A9-A15. Lane 7 and 8: AcrA-SP4, AKTA fractions B1-B5. M: molecular weight marker PageRuler Plus. Serotype 4 antiserum cross-reacts with AcrA. Only sample from lanes 2, 4 and 6 were used in vaccination experiments.
